# Supplementary material for: Does our legal minimum drinking age modulate risk of first heavy drinking episode soon after drinking onset? Epidemiological evidence for the United States, 2006–2014
Source: PeerJ. 2016 Jun 23;4:e2153. doi: 10.7717/peerj.2153 (PMC4924122; doi:10.7717/peerj.2153)
Supplement: Supplemental Information 1 [file peerj-04-2153-s001.docx]

**Supplementary material**

| **Supplementary Table S1. Number of Participants in Each Sex- and Age-Group. Data From the United States National Surveys on Drug Use and Health, 2006-2014 (Unweighted n = 24,100 12-to-23-Year-Olds).** | | | | | | | | | | | | |
| --- | --- | --- | --- | --- | --- | --- | --- | --- | --- | --- | --- | --- |
|  | **Age at First Drinking** | | | | | | | | | | | |
| **Year** | **Age=12** | **Age=13** | **Age=14** | **Age=15** | **Age=16** | **Age=17** | **Age=18** | **Age=19** | **Age=20** | **Age=21** | **Age=22** | **Age=23** |
| 2006 | 119 | 248 | 377 | 482 | 418 | 336 | 301 | 116 | 95 | 166 | 27 | 6 |
| 2007 | 110 | 227 | 387 | 462 | 458 | 323 | 312 | 104 | 89 | 220 | 24 | 17 |
| 2008 | 97 | 196 | 356 | 496 | 405 | 340 | 308 | 144 | 84 | 220 | 24 | 18 |
| 2009 | 98 | 230 | 338 | 475 | 436 | 333 | 326 | 134 | 85 | 240 | 22 | 14 |
| 2010 | 93 | 219 | 357 | 517 | 441 | 356 | 322 | 153 | 116 | 276 | 28 | 14 |
| 2011 | 85 | 237 | 353 | 506 | 498 | 355 | 312 | 137 | 96 | 303 | 29 | 19 |
| 2012 | 104 | 160 | 346 | 426 | 428 | 302 | 294 | 142 | 105 | 321 | 45 | 9 |
| 2013 | 66 | 165 | 337 | 437 | 465 | 321 | 318 | 121 | 117 | 307 | 30 | 13 |
| 2014 | 45 | 123 | 246 | 347 | 350 | 256 | 242 | 88 | 96 | 237 | 16 | 10 |

| **Supplementary Table S2. Age-, Cohort-, and Time-Specific Population Incidence Rate Estimates for Newly Incident Heavy Episodic Drinking. Data From United States National Surveys on Drug Use And Health 2006-2014 (Unweighted n=241,278 12-25 Year Olds).^a^** | | | | | | | | | | | | | |
| --- | --- | --- | --- | --- | --- | --- | --- | --- | --- | --- | --- | --- | --- |
|  | **Age=12** | | **13** | **14** | **15** | **16** | **17** | **18** | **19** | **20** | **21** | **22-23** | **24-25** |
| **Year** | | **Panel A. Estimated incidence of heavy episodic drinking among females** | | | | | | | | | | | |
| **2006** | | 0.8 | 1.8 | 6.0 | 11.2 | 17.7 | 19.8 | 26.4 | 25.4 | 25.4 | 35.2 | 19.8 | 10.7 |
| **2007** | | 0.2 | 1.8 | 4.2 | 10.5 | 16.8 | 18.6 | 25.9 | 23.5 | 24.2 | 35.7 | 16.1 | 10.0 |
| **2008** | | 0.2 | 1.0 | 4.8 | 8.5 | 16.0 | 18.8 | 22.2 | 23.7 | 18.3 | 34.5 | 16.8 | 9.2 |
| **2009** | | 0.9 | 2.6 | 5.3 | 9.4 | 15.9 | 16.9 | 25.1 | 25.5 | 20.1 | 29.3 | 19.3 | 8.0 |
| **2010** | | 0.1 | 2.3 | 3.5 | 9.3 | 11.9 | 16.5 | 22.9 | 22.9 | 24.4 | 34.5 | 20.2 | 9.9 |
| **2011** | | 0.5 | 1.2 | 3.2 | 7.4 | 10.8 | 20.4 | 21.7 | 19.2 | 16.8 | 35.5 | 21.2 | 8.5 |
| **2012** | | 0.3 | 0.7 | 4.5 | 6.8 | 13.8 | 17.9 | 22.4 | 22.0 | 22.9 | 28.9 | 16.8 | 8.9 |
| **2013** | | 0.3 | 1.1 | 2.7 | 7.6 | 9.4 | 14.7 | 16.8 | 20.8 | 22.5 | 31.7 | 15.8 | 8.7 |
| **2014** | | 0.1 | 1.9 | 3.3 | 7.2 | 11.3 | 13.0 | 18.1 | 19.1 | 16.9 | 30.0 | 18.6 | 10.0 |
|  | | | | | | | | | | | | | |
| M**eta-analysis estimates** ^b^ | | 0.2 | 1.5 | 3.9 | 8.6 | 13.7 | 18.1 | 22.6 | 22.6 | 21.7 | 33.6 | 18.2 | 9.4 |
| **Year** | | **Panel B. Estimated incidence of heavy episodic drinking among males** | | | | | | | | | | | |
| **2006** | | 0.3 | 2.8 | 9.4 | 12.5 | 16.8 | 16.5 | 21.3 | 14.8 | 15.7 | 30.7 | 14.7 | 7.3 |
| **2007** | | 0.7 | 1.7 | 5.7 | 12.5 | 14.8 | 16.1 | 20.4 | 19.9 | 14.3 | 27.2 | 11.9 | 7.3 |
| **2008** | | 1.1 | 2.6 | 4.9 | 11.4 | 14.9 | 14.7 | 22.3 | 19.2 | 14.8 | 27.7 | 12.0 | 8.1 |
| **2009** | | 0.7 | 2.9 | 5.0 | 9.6 | 13.4 | 14.4 | 19.1 | 17.0 | 15.7 | 30.3 | 13.4 | 6.0 |
| **2010** | | 0.1 | 1.7 | 5.0 | 10.5 | 13.5 | 12.3 | 19.7 | 17.2 | 14.6 | 26.1 | 14.6 | 7.4 |
| **2011** | | 0.8 | 1.8 | 4.1 | 10.9 | 13.5 | 12.6 | 18.4 | 20.5 | 17.1 | 27.1 | 16.1 | 4.8 |
| **2012** | | 0.5 | 1.6 | 4.9 | 9.8 | 12.4 | 14.5 | 18.2 | 19.2 | 16.6 | 30.1 | 14.1 | 6.7 |
| **2013** | | 0.2 | 0.7 | 3.2 | 6.8 | 9.7 | 13.6 | 15.9 | 21.3 | 15.7 | 30.8 | 13.6 | 6.7 |
| **2014** | | 0.1 | 0.9 | 2.0 | 6.6 | 12.5 | 19.2 | 17.3 | 21.3 | 23.5 | 36.1 | 19.4 | 10.6 |
|  | | | | | | | | | | | | | |
| **Meta-analysis estimates** ^b^ | | 0.4 | 2.0 | 4.9 | 10.0 | 13.4 | 14.3 | 19.4 | 18.7 | 15.6 | 28.7 | 13.9 | 6.9 |
| **^a.^** Adjacent cells with the same shade trace the experience of individual cohorts.  ^b.^ Meta-analysis summary estimates with each year treated as an independent replication. For 12-to-16-year-olds, heterogeneity across replications motivated use of the random effects variance estimation approach. | | | | | | | | | | | | | |

| **Supplementary Table S3. 95% Confidence Intervals of Age-, Cohort-, and Time-Specific Population Incidence Rate Estimates for Newly Incident Heavy Episodic Drinking. Data From United States National Surveys on Drug Use And Health 2006-2013 (Unweighted n=241,278 12-25 Year Olds).^a^** | | | | | | | | | | | | |
| --- | --- | --- | --- | --- | --- | --- | --- | --- | --- | --- | --- | --- |
|  | Age=12 | 13 | 14 | 15 | 16 | 17 | 18 | 19 | 20 | 21 | 22-23 | 24-25 |
| Year | **Panel A. 95% Confidence Interval of Estimated Incidence of Heavy Episodic Drinking among Females** | | | | | | | | | | | |
| 2006 | 0.4,1.7 | 1.2,2.9 | 4.7,7.5 | 9.1,13.6 | 15.1,20.6 | 17.5,22.3 | 22.3,31.0 | 20.5,30.9 | 19.9,31.9 | 30.0,40.8 | 15.9,24.6 | 7.8,14.7 |
| 2007 | 0.0,0.6 | 1.2,2.8 | 3.2,5.3 | 8.7,12.6 | 14.8,18.9 | 15.9,21.7 | 21.9,30.5 | 19.5,28.0 | 19.4,29.8 | 29.6,42.3 | 12.4,20.7 | 7.3,13.7 |
| 2008 | 0.1,0.6 | 0.5,1.9 | 3.7,6.2 | 7.0,10.4 | 13.6,18.8 | 15.6,22.5 | 19.0,25.7 | 19.4,28.7 | 14.1,23.3 | 28.8,40.6 | 12.9,21.6 | 5.8,14.2 |
| 2009 | 0.4,2.2 | 1.5,4.3 | 4.1,7.0 | 7.6,11.5 | 13.8,18.3 | 14.4,19.8 | 21.3,29.4 | 20.4,31.3 | 16.3,24.5 | 24.0,35.3 | 15.6,23.5 | 5.7,11.3 |
| 2010 | 0.0,0.3 | 1.4,3.9 | 2.5,4.9 | 7.4,11.7 | 10.0,14.2 | 14.4,18.8 | 19.5,26.8 | 18.3,28.3 | 20.1,29.3 | 29.0,40.3 | 15.8,25.4 | 6.5,14.9 |
| 2011 | 0.2,1.4 | 0.7,2.0 | 2.3,4.4 | 6.0,9.2 | 8.5,13.6 | 17.9,23.1 | 18.2,25.6 | 15.4,23.8 | 13.8,20.3 | 29.4,42.0 | 16.9,26.4 | 5.8,12.2 |
| 2012 | 0.0,1.7 | 0.3,1.9 | 3.2,6.2 | 5.6,8.3 | 11.7,16.2 | 14.9,21.3 | 18.7,26.6 | 17.7,26.9 | 17.4,29.5 | 24.0,34.3 | 14.1,20.0 | 6.3,12.4 |
| 2013 | 0.1,0.9 | 0.6,2.0 | 1.9,3.9 | 6.1,9.6 | 7.7,11.3 | 12.7,16.9 | 13.9,20.2 | 17.7,24.2 | 17.5,28.4 | 25.9,38.1 | 12.6,19.5 | 5.6,13.2 |
| 2014 | 0.2, 0.7 | 1.3, 2.1 | 3.7, 5.3 | 8.0, 10.4 | 12.5, 16.6 | 17.1, 18.9 | 21.6, 24.2 | 21.3, 24.3 | 19.5, 27.0 | 30.6, 34.3 | 16.6, 19.3 | 7.9, 10.1 |
|  | | | | | | | | | | | | |
| **Meta-analysis estimates** ^b^ | 0.1, 0.5 | 1.1, 1.9 | 3.2, 4.8 | 7.6, 9.7 | 12.0, 15.7 | 17.2, 19.0 | 21.4, 23.9 | 21.2, 24.1 | 20.2, 23.3 | 31.8, 35.5 | 17.0, 19.6 | 8.3, 10.6 |
| Year | **Panel B. 95% Confidence Interval of Estimated Incidence of Heavy Episodic Drinking among Males** | | | | | | | | | | | |
| 2006 | 0.1,1.0 | 1.8,4.4 | 7.2,12.1 | 10.4,14.9 | 14.5,19.4 | 14.1,19.1 | 18.1,25.0 | 11.7,18.7 | 12.1,20.1 | 26.5,35.3 | 12.0,17.8 | 5.6,9.4 |
| 2007 | 0.3,1.5 | 1.0,2.8 | 4.2,7.7 | 10.2,15.2 | 12.5,17.4 | 13.1,19.6 | 16.6,24.9 | 16.2,24.3 | 11.1,18.3 | 23.1,31.7 | 10.0,14.1 | 5.6,9.5 |
| 2008 | 0.5,2.3 | 1.8,3.8 | 3.7,6.5 | 9.1,14.2 | 12.5,17.7 | 12.3,17.5 | 18.4,26.8 | 15.8,23.1 | 11.6,18.7 | 23.7,32.2 | 9.3,15.3 | 5.5,11.5 |
| 2009 | 0.2,2.3 | 1.8,4.6 | 3.9,6.5 | 7.7,11.8 | 11.3,15.8 | 11.6,17.8 | 16.1,22.5 | 13.8,20.8 | 12.4,19.7 | 25.8,35.2 | 10.9,16.4 | 4.6,7.8 |
| 2010 | 0.0,0.3 | 1.1,2.5 | 3.9,6.4 | 8.4,13.0 | 11.3,16.0 | 9.8,15.5 | 16.3,23.5 | 14.3,20.7 | 11.6,18.2 | 21.7,31.0 | 12.4,17.0 | 5.3,10.2 |
| 2011 | 0.2,3.1 | 1.1,2.8 | 3.1,5.5 | 9.1,13.1 | 11.1,16.3 | 10.5,15.0 | 16.0,21.2 | 17.0,24.6 | 13.3,21.6 | 22.7,32.1 | 13.0,19.7 | 3.4,6.7 |
| 2012 | 0.2,1.2 | 0.9,2.9 | 3.0,7.6 | 7.7,12.4 | 10.1,15.1 | 11.8,17.8 | 14.5,22.6 | 15.4,23.8 | 12.9,21.1 | 25.1,35.5 | 11.6,17.2 | 5.2,8.6 |
| 2013 | 0.1,1.0 | 1.8,4.4 | 7.2,12.1 | 10.4,14.9 | 14.5,19.4 | 14.1,19.1 | 18.1,25.0 | 11.7,18.7 | 12.1,20.1 | 26.5,35.3 | 12.0,17.8 | 5.6,9.4 |
| 2014 | 0.3, 1.0 | 1.8, 2.5 | 4.4, 6.5 | 9.2, 11.9 | 12.3, 15.0 | 13.6, 15.9 | 18.3, 20.7 | 17.4, 19.9 | 13.8, 20.5 | 26.0, 30.2 | 12.7, 14.5 | 6.0, 7.2 |
|  | | | | | | | | | | | | |
| **Meta-analysis estimates** ^b^ | 0.3, 0.7 | 1.7, 2.3 | 4.0, 6.1 | 8.7, 11.5 | 12.0, 14.8 | 13.3, 15.3 | 18.2, 20.6 | 17.5, 20.0 | 14.4, 17.0 | 27.1, 30.3 | 13.0, 14.9 | 6.2, 7.7 |
| **^a.^** Adjacent cells with the same shade trace the experience of individual cohorts.  ^b.^ Meta-analysis summary estimates with each year treated as an independent replication. For 12-to-16-year-olds, heterogeneity across replications motivated use of the random effects variance estimation approach. | | | | | | | | | | | | |

| **Supplementary Figure S1. Meta-analytic Summary Estimates for Sex- and Age-Specific Population Incidence Rates for 1^st^ Heavy Episodic Drinking. Data from United States National Surveys on Drug Use and Health, 2006-2014 (unweighted n=241,278 12-25 year olds).** ^a^ |
| --- |
|  |
| ^a.^ For 14 and 20-to-25-year-old males as well as 18-25-year-old females, heterogeneity across replications motivated use of the random effects variance estimation approach. |
